# Assessing cohesion and diversity in the collaboration network of the SALURBAL project

**DOI:** 10.1038/s41598-023-33641-x

**Published:** 2023-05-10

**Authors:** Sofía Baquero, Felipe Montes, Ivana Stankov, Olga L. Sarmiento, Pablo Medina, S. Claire Slesinski, Francisco Diez-Canseco, Maria F. Kroker-Lobos, Waleska Teixeira Caiaffa, Alejandra Vives, Marcio Alazraqui, Tonatiuh Barrientos-Gutiérrez, Ana V. Diez Roux

**Affiliations:** 1 Department of Industrial Engineering, Social and Health Complexity Center, Universidad de los Andes, Crr 1 Este No.19ª-40 Piso 8, 111711 Bogotá, Colombia; 2 Urban Health Collaborative, Dornsife School of Public Health, Drexel University, Philadelphia, PA 19104 USA; 3 UniSA Allied Health and Human Performance, University of South Australia, Adelaide, 5000 Australia; 4 School of Medicine, Universidad de los Andes, 111711 Bogotá, Colombia; 5 Pettenkofer School of Public Health, Ludwig-Maximilians-Universität München, 81377 Munich, Germany; 6 CRONICAS Centre of Excellence in Chronic Diseases, Universidad Peruana Cayetano Heredia, Lima, 15074 Peru; 7 INCAP Research Center for the Prevention of Chronic Diseases (CIIPEC), Institute of Nutrition of Central America and Panama (INCAP), Guatemala City, 01011 Guatemala; 8 Observatory for Urban Health in Belo Horizonte (OSUBH), Universidade Federal de Minas Gerais, Brazil, Belo Horizonte, MG 30130-100 Brazil; 9 Department of Public Health, CEDEUS, Universidad Católica de Chile, 8330077 Santiago, Chile; 10 Institute of Collective Health, National University of Lanús, Buenos Aires, Argentina; 11 Center for Population Health Research, National Institute of Public Health, 62100 Cuernavaca, Mexico

**Keywords:** Complex networks, Translational research

## Abstract

The SALURBAL (Urban Health in Latin America) Project is an interdisciplinary multinational network aimed at generating and disseminating actionable evidence on the drivers of health in cities of Latin America. We conducted a temporal multilayer network analysis where we measured cohesion over time using network structural properties and assessed diversity *within* and *between* different project activities according to participant attributes. Between 2017 and 2020 the SALURBAL network comprised 395 participants across 26 countries, 23 disciplines, and 181 institutions. While the cohesion of the SALURBAL network fluctuated over time, overall, an increase was observed from the first to the last time point of our analysis (clustering coefficient increased [0.83–0.91] and shortest path decreased [1.70–1.68]). SALURBAL also exhibited balanced overall diversity *within* project activities (0.5–0.6) by designing activities for different purposes such as capacity building, team-building, research, and dissemination. The network’s growth was facilitated by the creation of new diverse collaborations across a range of activities over time, while maintaining the diversity of existing collaborations (0.69–0.75 between activity diversity depending on the attribute). The SALURBAL experience can serve as an example for multinational research projects aiming to build cohesive networks while leveraging heterogeneity in countries, disciplines, career stage, and across sectors.

## Introduction

Over the past five decades, collaboration has played an increasingly important role in the production of knowledge and in scientific innovation^1^. Science to improve urban health is no exception, as multiple perspectives and disciplines are key to scientific understanding and to the identification of effective actions. Multidisciplinary and geographically diverse collaborative networks can strengthen urban health inquiry by bringing together diverse ideas, knowledge, experiences, and strategies to illuminate complex problems and their potential solutions. Diverse collaborative networks have been recognized as drivers of value creation, pushing the boundaries of innovative and impactful research^2^. These networks also provide access to resources, including physical facilities and diverse funding streams, and can also facilitate capacity-building and innovation by exposing researchers to a wide range of disciplines, tools, and data^2,3^.

Research networks have been more limited in low- and middle-income countries than in the Global North^3^. The paucity of research networks facilitating collaboration between diverse stakeholders across the countries of Latin America may impact the continent’s progress towards meeting the United Nations Sustainable Development Goals (SDGs). For example, Latin America has made limited progress towards achieving SDG 9, which focuses on innovation, research, and development^4^. Latin America and the Caribbean are far behind other world regions in terms of research and development. Latin American countries have invested only 0.71% of their Gross Domestic Product (GDP) in research and technology, compared to investments ranging from 2.8 to 5% of GDP in the United States, Japan, and the Republic of Korea^5^. This lack of investment can undoubtedly impact the production of locally and globally relevant research in the region and has limited capacity-building opportunities available to junior researchers and practitioners. Fostering scientific innovation and development within Latin America necessitates the creation of an interdisciplinary and internationally diverse research environment that encourages the development of scientists, institutions, and collaborative networks^6^.

One recent example of an innovative multi-institutional scientific collaboration across Latin America is the SALURBAL (Salud Urbana en América Latina, or Urban Health in Latin America) Project. Established in 2017, SALURBAL was created with the overall goal of generating actionable evidence to inform policies and interventions that create healthier, more equitable, and sustainable cities in Latin America^7^. The project’s organizational and governance structures^7^ were designed to engage a large, interdisciplinary team spanning several Latin American countries, with participants from different sectors and levels of career stage. Important accomplishments of SALURBAL to date include: (1) generating the largest compilation, harmonization and geographic linkage of mortality and survey data with environmental indicators at different scales for all cities (n = 371) of 100,000 residents or more in 11 countries of Latin America, (2) evaluations of transformative urban policies and their varied impacts on health, (3) the application of systems thinking to better understand policy impacts on urban health in the region, (4) capacity building of junior and senior researchers in Latin America on urban health methodologies, and (5) promoting and disseminating actionable evidence on the drivers of health in cities in Latin America. However, the collaborative networks that have evolved as part of this groundbreaking project and that serve as the foundation for the projects’ diverse outputs, remain undocumented.

Network analysis is a powerful tool that can be used to characterize collaborations between individuals and groups. Two key features of networks that can be assessed and form important indicators of scientific collaboration include network cohesion and network diversity. Network cohesion is a measure of the connectedness and togetherness among members and of the degree to which these relationships are widely distributed within the network^8^. Network cohesion can facilitate the emergence of novel ideas^9,10^ and can play an important role in effective knowledge transfer and information diffusion^11^. Network diversity captures the extent to which network interactions occur between individuals with different attributes. In this study, we measure diversity in two ways: (a) the diversity of members (in terms of personal attributes) who collaborate on a given activity type, and (b) the diversity in the configuration of connections between members contributed by adding different types of activities to the project. Network diversity can foster productivity and innovation by bringing together individuals with different perspectives and skill sets, and can enhance the ability to grapple with complex research problems^12^. Diversity can also create logistical challenges in hiring and the distribution of funding and other resources, as well as obstacles in communication and coordination between different groups, with potentially conflicting values and priorities^12,13^. Leveraging diversity to maximize productivity, innovation, robustness and longevity of collaborative teams is an important goal for large collaborative projects^14^.

The aim of this study was to characterize the SALURBAL network and explore the extent to which the project promoted cohesiveness among network participants and diversity *within* and *between* project activities. We used temporal network analysis and novel cohesion and diversity measures to: (1) characterize the evolving cohesion of the SALURBAL network over the initial three years of the project (from 2017 to 2020), and (2) determine the diversity in collaborations across participant attributes (country, city, discipline, research topic, sector, career stage, gender) *within* and *between* project activities (proposals, academic workshops, meetings, group model building workshops, papers, forums and symposia) over time.

## Results

### Characterization of the SALURBAL collaboration network over time

**Figure 1:**
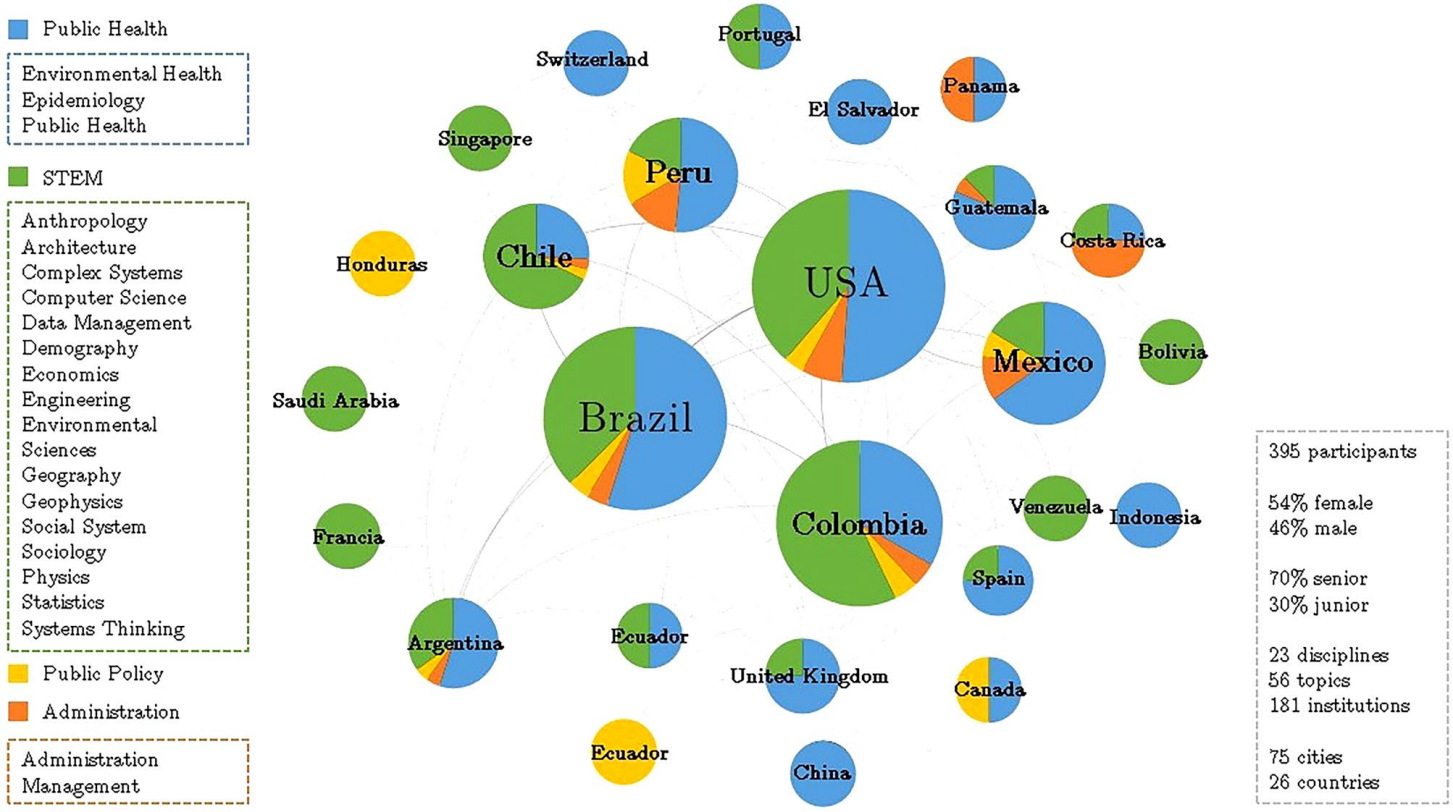
The SALURBAL interdisciplinary network by country. Each node represents a country that participated in the project. Node size represents the number of participants. Each node has a pie chart with the distribution of disciplines of its participants divided into fields for better visualization. The National Science Foundation's classification of STEM fields was used, except for STEM disciplines within public health that are included in public health.

**Figure 2:**
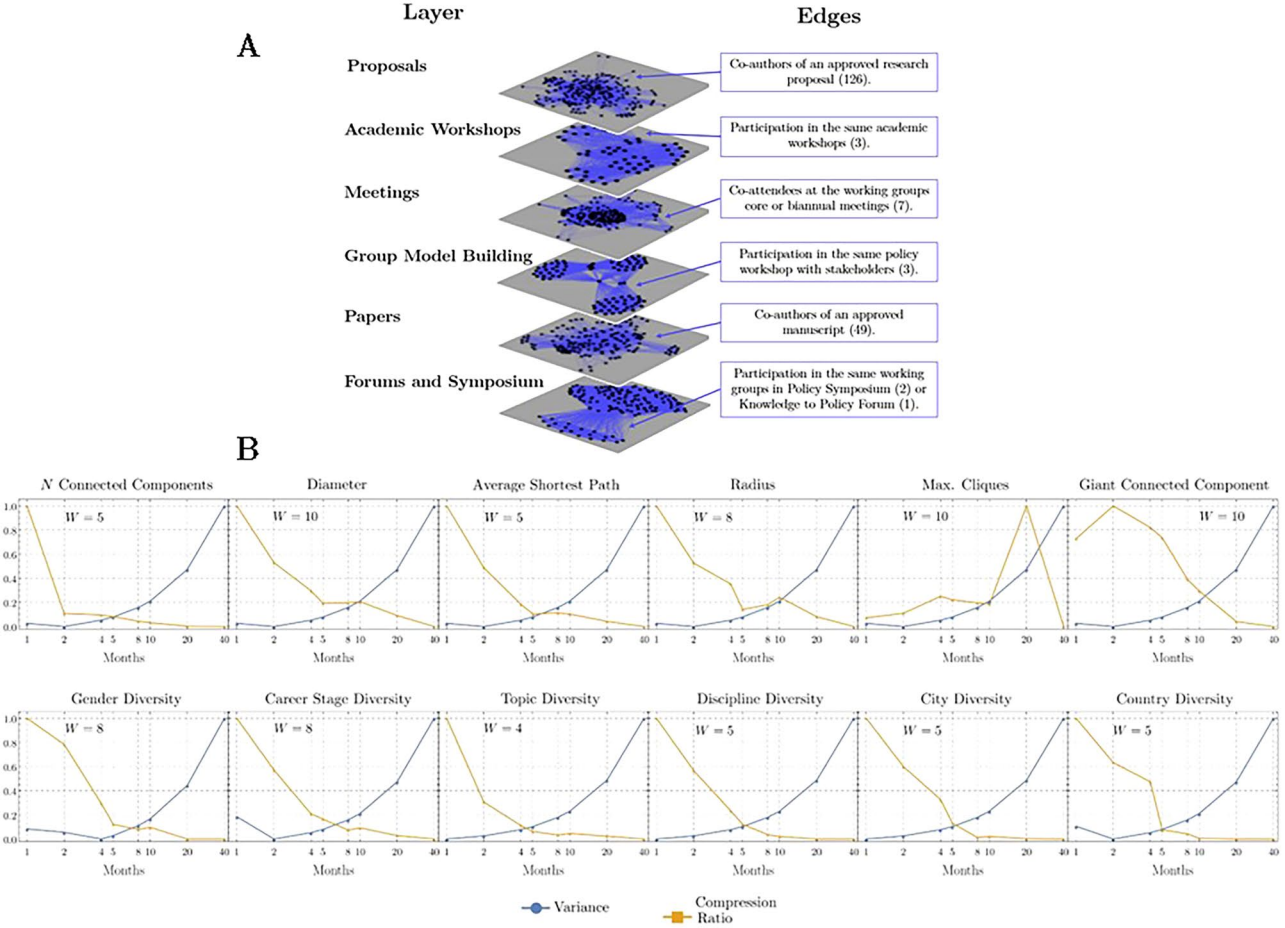
SALURBAL temporal multilayer network. (**A**) The optimal temporal window size w that minimizes the absolute difference between variance ${V(F}_{w}),$ and compression ratio $R\left({F}_{w}\right)$ for each network measure using the TWIN algorithm. (**B**) The multilayer network structure of the SALURBAL Project. The nodes represent SALURBAL participants and the edges represent collaborations between participants as defined by the text box corresponding to each layer.

Within the period of May 2017 to August 2020, the SALURBAL network included 395 participants (researchers, policymakers, and representatives from civil society organizations and non-governmental organizations) across 26 countries (16 from the Americas, five from Europe, four from Asia, and Australia), 73 cities, 23 disciplines, and 181 institutions (Fig. 1). Each participant (node) was characterized according to seven attributes (country, city, discipline, research topic, sector, career stage, gender). The edges between nodes represent collaborations between participants. The collaborations were classified into types according to six project activities. Each type of collaboration was represented as a layer in a multiplex network structure (Fig. 2A). Aggregated from May 2017 to August 2020, the layers of this network represent and encompass six different project activity types, including: (I) 126 research proposals with a total of 228 participants forming 2435 collaborative ties, (II) three academic training workshops with a total of 59 participants forming 1125 collaborative ties, (III) seven in-person team meetings in SALURBAL’s country hubs and virtual full team meetings, as well as virtual core and working groups meetings with a total of 226 participants forming 12,720 collaborative ties, (IV) three group model building (GMB) workshops with a total of 95 participants forming 1827 collaborative ties, (V) 49 papers published or approved by the SALUBAL publications committee involving a total of 158 participating coauthors forming 1805 collaborative ties; and (VI) two policy symposia, and one Knowledge to Policy Forum with a total of 147 participants forming 7631 collaborative ties. Given the diverse timing of activities across layers of the network, there was no intuitive way to split the network into time windows based on the timing of project activities. As such, we used the Temporal Window in Networks (TWIN) algorithm to determine the optimal window size for detecting changes in network diversity (see “Methods”). Informed by this algorithm, and for the purposes of the analysis, we divided the network into five time windows, each capturing a period of eight months (Fig. 2B).

### SALURBAL network cohesion over time

Cohesion was measured using three structural measures: the network density (the number of observed collaborations over the total number of all possible collaborations); the average clustering coefficient (the proportion of a given participant’s collaborators who are also connected (i.e., collaborate) with each other), and the average shortest path (the average number of steps along the shortest path for all possible collaborations between participants (i.e., the average number of people you will have to communicate through to contact a complete stranger))^15^. A cohesive network is dense, with a high clustering coefficient and a low average shortest path. A network with this path structure holds the network together because it has multiple paths between participants, resulting in high levels of information diffusion^8^. The SALURBAL aggregated network (from May 2017 to August 2020) showed a positive slope in cohesion over time, as reflected by increases in the average clustering coefficient ([0.83–0.91]; slope = 0.01), and an increase in network density ([0.34–0.42]; slope = 0.01) (Table 1). Cohesion rose in the first 15 months as the density grew from 0.34 to 0.44, the clustering coefficient increased from 0.83 to 0.94 while the shortest path decreased from 1.70 to 1.58. Then, in the subsequent eight months, the cohesion decreased (i.e., the clustering coefficient fell from 0.93 to 0.88 while the shortest path increased from 1.58 to 1.81). In the last 23 months (until August 2020), the cohesion of the network rose again as the density and clustering coefficient increased from 0.25 to 0.42 and from 0.88 to 0.91, respectively, and the shortest path decreased from 1.81 to 1.68.

**Table 1:** Structural properties of the SALURBAL collaboration aggregated network over time (2017–2020).

Periods of time	Density	Average clustering coefficient	Average shortest path
May 2017 to Dec 2017	0.34	0.83	1.70
Jan 2018 to Aug 2018	0.44	0.94	1.58
Sep 2018 to Apr 2019	0.25	0.88	1.81
May 2019 to Dec 2019	0.34	0.87	1.71
Jan 2020 to Aug 2020	0.42	0.91	1.68

### Diversity *within* layers (project activities) without temporal effects

**Figure 3:**
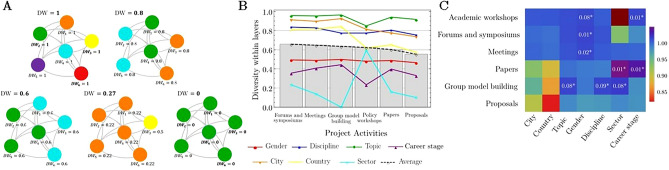
The diversity *within* layers (i.e., project activities). (**A**) Diversity *within* layers (DW) in five examples of working groups: (**A.1**) All the participants are from different countries, $\mathrm{DW}=1$. (**A.2**) One-third of the participants are from USA (blue nodes), another third are from Brazil (green nodes), and the rest are from Mexico (orange nodes), $\mathrm{DW}=0.8$. (**A.3**) Half of the participants are from Brazil and the other half from USA, $\mathrm{DW}=0.6.$ (**A.4**) One participant is from Colombia (yellow nodes), the rest are from Mexico, $\mathrm{DW}=0.27.$ (**A.5**) All the participants are from Brazil, $\mathrm{DW}=0$. (**B**) The diversity *within* the SALURBAL Project activities. Each line represents the diversity of each attribute, by project activity. Each bar graph represents the overall diversity within a particular activity, i.e. the average diversity across all attributes. The bars are in order of highest to lowest average diversity of each activity. (**C**) The ratio of the SALURBAL diversity to the simulated diversity on 1000 randomly generated networks that preserve the degree sequence of the participants' attributes of the SALURBAL network. Each panel represents a project activity and an attribute diversity. The purple indicates a higher diversity than expected by chance. *The p-value < 0.1 indicates that there are significantly more diverse collaborations than expected by chance.

We measured the diversity *within* each layer (i.e., project activity) across seven participant attributes. This diversity score ranges from zero to one (see “Methods”). Larger values represent a higher proportion of collaborations between a given individual and participants with different attributes encompassing both dyadic and working group level connections (Fig. 3A). Overall, we found that the average diversity across all attributes was similar *within* each project activity type and ranged from 0.5 to 0.6 (Fig. 3B). When disaggregating the diversity score by attribute, the SALURBAL network was characterized by high diversity *within* all activities by country (0.72), city (0.84), discipline (0.79), and research topic (0.93). Higher diversity was observed in the geographical attributes for the academic training workshops (country = 0.84, city = 0.92), meetings (country = 0.84, city = 0.92), forums and symposia (country = 0.81, city = 0.91). The academic training workshops were characterized by the highest diversity in career stage (0.44) while the GMB workshops had the highest sector diversity (0.59) compared to the other activities (Fig. 3B).

For each layer and attribute, we undertook a validation process to discern whether the observed diversity was an intrinsic property of the network or whether it could be attributed purely to chance. To do this, we implemented a degree-constrained configuration model which compared the diversity observed for each type of activity and attribute in the project network with diversity that would emerge from 1000 randomly generated networks^16^ (“Methods”). We observed that senior participants were more likely to collaborate with junior participants during academic training workshops and co-author papers than would be expected by chance (p-value = 0.01). Moreover, intersectoral collaborations during GMB workshops and on academic papers were more likely than expected by chance (p-value = 0.01 and 0.08, respectively). The discipline and research topic diversities were higher than expected by chance for the GMB workshops (p-value = 0.09 and 0.08, respectively). Forums, meetings, and academic training workshops were characterized by higher gender diversity than would be expected by chance, with p-values of 0.01, 0.02, and 0.08, respectively (Fig. 3C).

### Diversity *between* layers (project activities) without temporal effects

**Figure 4:**
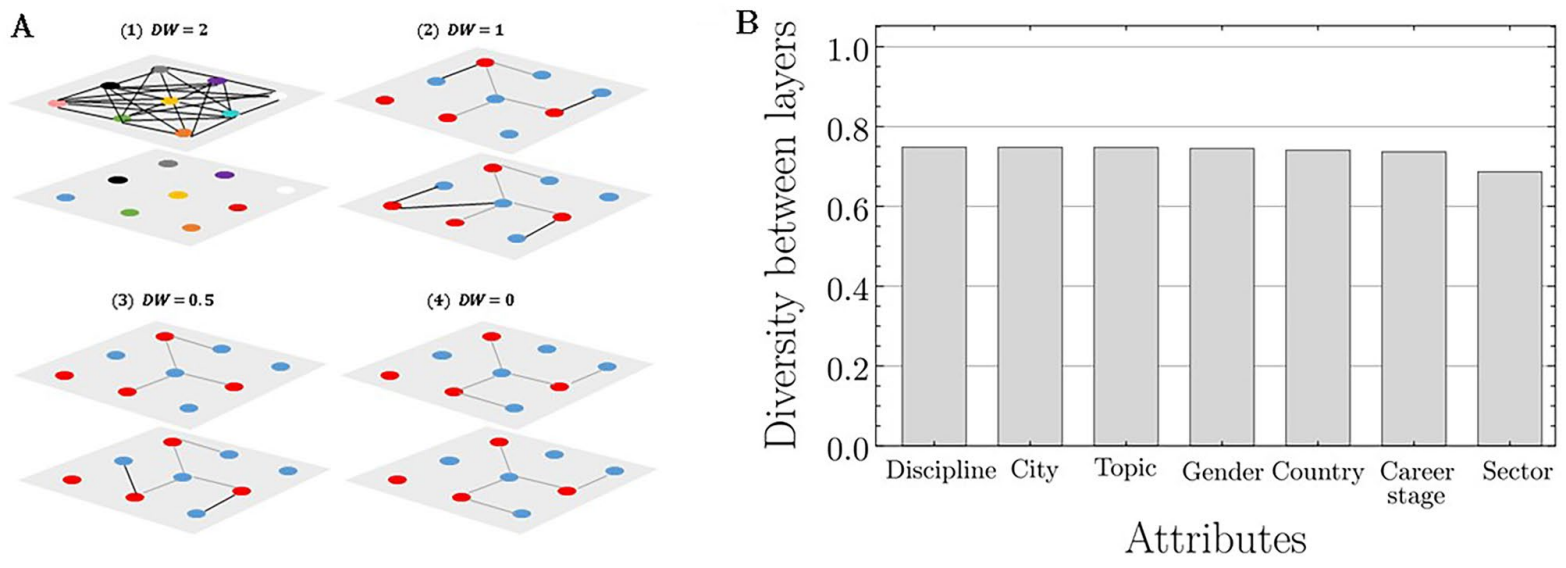
The diversity *between* layers (i.e., project activities). (**A**) Conceptual representation of the diversity *between* layers (DB) measure using four examples. Each layer represents an activity, each node represents a person in the project (red = female, blue = male) and the edges (or connections between nodes) represent collaborations between people. (**A.1**) In the most extreme and special case, where two layers are maximally different $(DB=2)$, the nodes in one layer are completely disconnected (i.e., no collaborations exist for that activity), while the other layer is completely connected (i.e., every person is collaborating with every other person, and each person is different from every other person with respect to a given attribute (nodes are all different colors)). (**A.2**) Half of the diverse collaborations between people are unique (black edges) while the other half are the same in both activities (grey edges) $(DB=1)$. (**A.3**) Most of the diverse collaborations are the same in both activities (gray edges) and there are very few collaborations that are unique to one activity and not the other (black edges) $(DB=0.5)$. (**A.4**) The diversity between layers is zero $(DB=0)$, that is, all the diverse collaborations are the same in both activities (gray edges). (**B**) The global diversity *between* layers for each attribute in the SALURBAL network, ordered from highest to lowest diversity *between* layers. The global diversity score for each attribute was calculated by evaluating the diversity *between* every possible pair of project activities, for a given attribute, and then aggregating this information into a single score using the layer reduction method.

The diversity *within* layers characterizes the proportion of collaborations that exist between individuals with different attributes within a specific activity. The diversity *between* layers is calculated separately for each of the seven attributes and characterizes differences in connectivity between activity layers. For a given attribute, the diversity *between* layers score measures the extent to which a given layer contributes new diverse collaborations. In other words, it measures the extent to which the collaborations between diverse participants for a given activity are unique from the collaborations that exist for another activity. A global diversity score for each attribute can calculated by evaluating the diversity between every possible pair of project activities, and then aggregating this information into a single score (see Fig. 4 and “Methods”). Diversity *between* layers ranges from zero to two. A value of two means that two layers are maximally different. That is, the nodes in one layer are completely disconnected (i.e., no collaborations exist for that activity), while the other layer is completely connected (i.e., every person is collaborating with every other person). A score of one indicates that for a given attribute, any given pair of activities include around half unique collaborations, and half that are the same people collaborating in both activities. On the other hand, a score of zero indicates that exactly the same people are collaborating in both activities. We found that SALUBRAL project activities overall, contributed similarly to *between* layer diversity of each attribute. The diversity *between* layers scores ranged from 0.69 for sector to around 0.75 for discipline, research topic, city, country, career stage, and gender (Fig. 4B). Aggregated across all attributes, we found that the SALURBAL Project network had a mean diversity *between* layers score of 0.74 (Fig. 4B), which indicates that almost half the diverse collaborations were new and unique, while the rest comprised pairs collaborating together across multiple project activities.

**Figure 5:**
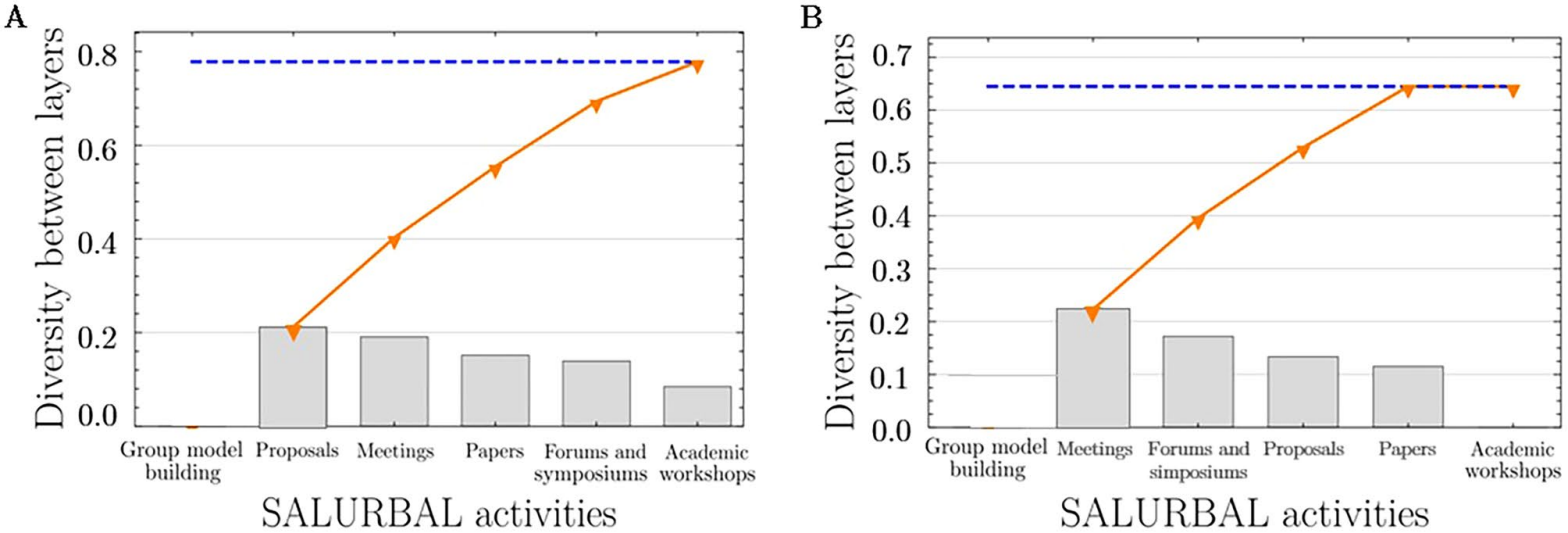
Shows, in descending order, the diversity contribution of each activity (bars) towards the diversity *between* layers for two attributes: discipline (**A**) and sector (**B**). The orange line represents the cumulative diversity *between* layers as it grows with the contribution of each project activity. The blue dash line shows the global diversity *between* layers for that attribute. The diversity contribution of each activity (i.e., each bar) is calculated through a series of pairwise comparisons between that activity, and each activity to its left. For example, in figure A, the diversity contribution of papers is determined by making pairwise comparisons between papers and meetings, papers and proposals and papers and group-model building activities, and then aggregating this information into a single score using the layer reduction method. (**A**) Group model building, and proposals are the largest contributors to *between* layer diversity in discipline by bringing the 27% of the global diversity, while (**B**) Group model building, and meetings contribute most to *between* layer diversity across sector by bringing the 35% of the global diversity. Note: the remaining attributes, including, gender, country, city, career stage, and research topic are not depicted as the contribution of each activity towards the diversity *between* layers for these attributes closely resembles the patterns observed for discipline, shown in figure (**A**).

We implemented a layer reduction method, which eliminates activities according to their contribution to global diversity, in order to identify the activities that contribute most to the global diversity *between* activities (“Methods”). The activity that added the greatest new diversity collaborations was the GMB workshops where researchers engaged with non-academic partners (Fig. 5). The activity that added the least new diverse collaborations were the academic training workshops (0.21) (Fig. 5).

### Assessing the diversity of the project over time

**Figure 6:**
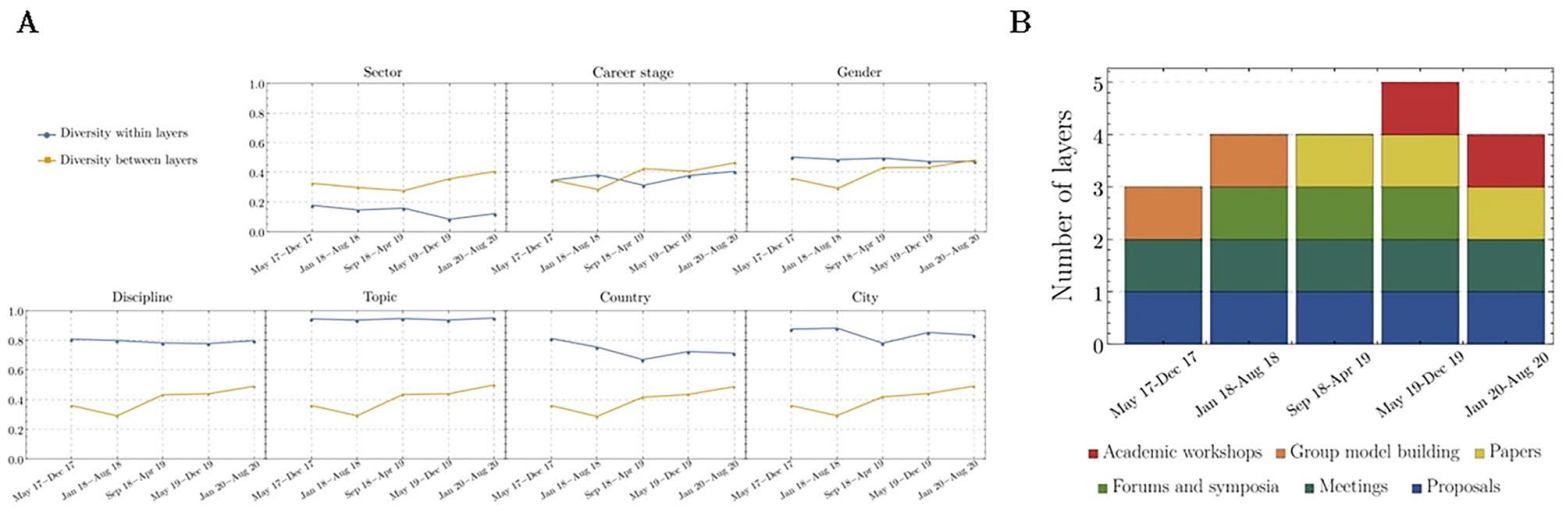
SALURBAL Project diversity over time (2017–2020). (**A**) Diversity within (orange line) and between (blue line) layers for each attribute over time. (**B**) Distribution of the project activities over time.

**Figure 7:**
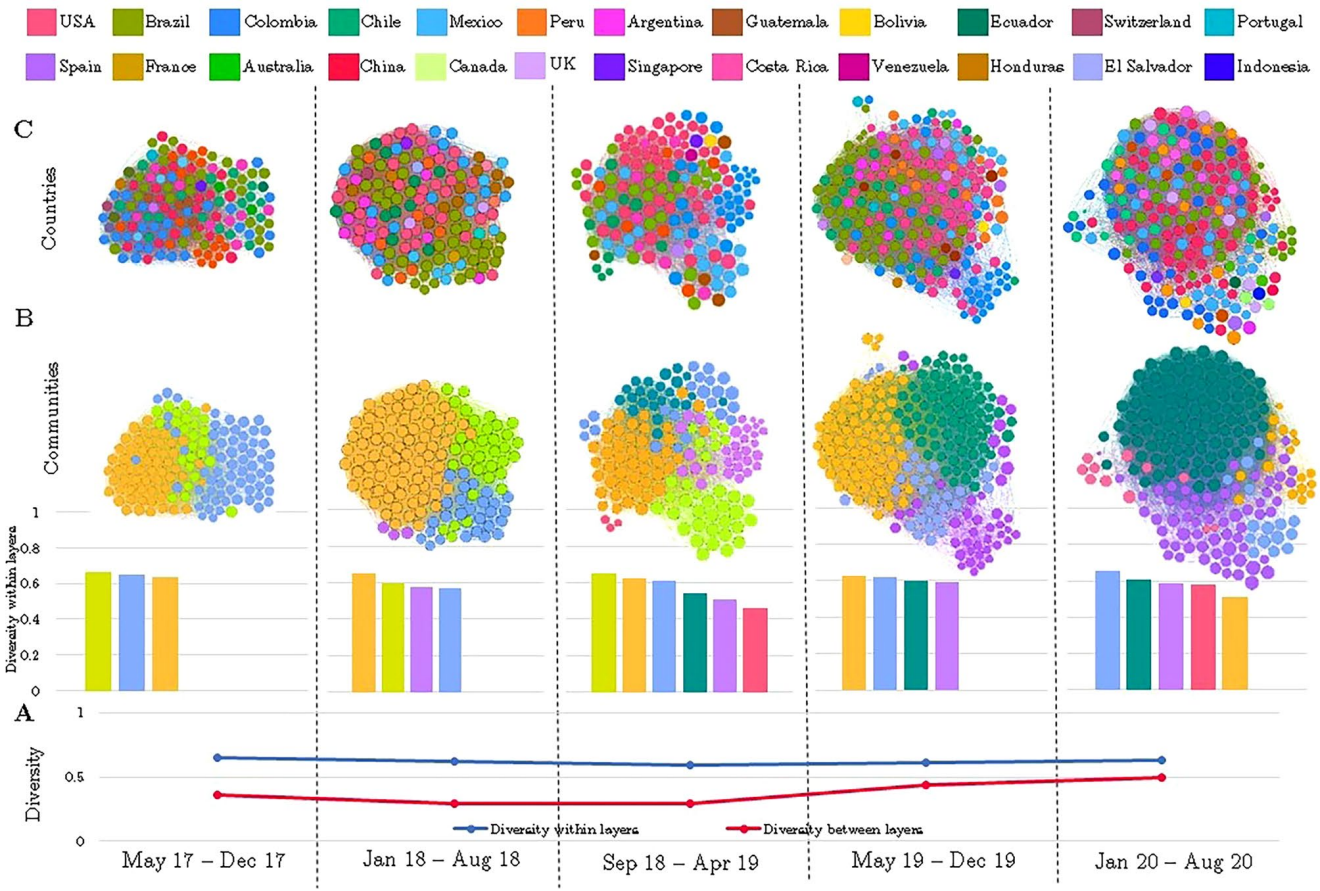
SALURBAL collaboration network over time (2017–2020). Each node represents a project participant. Node size represents the diversity of the participant's collaborations, the smaller the node, the lower the diversity. (**A**) The average diversity *within* layers of all the SALURBAL activities vs the global diversity *between* layers over time. (**B**) Diversity within communities over time, where the node color represents the participant´s community. The communities are the groups of participants that are highly interconnected, as defined by the Louvain method, compared to the rest of participants. Each bar graph represents the community´s diversity by averaging across all attributes. (**C**) SALURBAL network where each node color represents the participant´s country.

We calculated the diversity *within* and *between* layers for each attribute over time. The diversity *within* layers for each attribute, specifically, discipline, research topic and gender, was quite stable over time with a standard deviation lower than 0.01 (Fig. 6A). In contrast, the diversity *within* layers for each of the other attributes, namely, sector, career stage, and geographic location, had a standard deviation greater than 0.03 over time (Fig. 6A). This standard deviation appeared to be associated with the number of different activities carried out in each temporal window (Fig. 6B). To test the extent to which each project activity was associated with diversity *within* and *between* layers, we implemented a multiple linear regression with categorical variables (where, the dependent variable is the average of the diversity *within* and *between* layers, and the independent variables are binary variables for each project activity, that is equal to one if the activity was realized in that time window or zero, otherwise). This model showed that academic training workshops (p-value = 0.02), forums and symposia (p-value = 0.05) and GMB workshops (p-value = 0.08) were statistically significant in generating diversity *within* and *between* activities in the collaborations over time. The SALURBAL diversity *within* layers were almost balanced and constant over time, with a mean of 0.62 (Fig. 7A). The diversity *between* layers increased over time with a range of 0.36–0.50 (Fig. 7A).

Furthermore, we identified the communities of participants that are highly interconnected throughout the aggregated network of the SALURBAL Project using the Louvain community detection algorithm. These communities are groups of participants where the connections between them are denser than connections with the rest of the network^16^. The diversity *within* these communities was similar in the periods May 2017 to December 2017 and May 2019 to December 2019 (Fig. 7B). Between these two periods (i.e., from January 2018 to April 2019) the communities were characterized by lower levels of geographic and sector diversity. Lastly, in the period of January 2020 to August 2020 smaller communities including participants from just a few different countries were formed (Fig. 7C). Overall, the communities are constituted by diverse participants, all communities were characterized by a mean diversity score higher than 0.5, with a mean of 0.6 (Fig. 7B).

## Discussion

The findings of our network analysis suggest that the SALURBAL Project network is both cohesive and diverse. Moreover, the diversity of collaborations between participants and the cohesion of the network have increased over time. By August 2020, the SALURBAL network evolved to include 395 participants across 26 countries, 24 disciplines, 171 institutions, numerous sectors, and career stage levels focused on urban health research, capacity-building, and knowledge translation. The collaborations within this network have largely included a balanced representation of members by gender and across levels of career stage. These findings affirm that SALURBAL’s organizational structure and activities succeeded in affording numerous avenues for diverse participants to meet and establish collaborative partnerships, across a range of project activities, and achieving a cohesive growing network over time. This balance between network diversity and cohesion represents a unique strength of the SALURBAL Project, as network diversity and cohesion are likely to be important to knowledge generation, dissemination, and capacity building.

Our findings indicate that the governance and administrative structures of the SALURBAL project supported the creation of a cohesive collaboration network. The cohesiveness of the network increased from its inception until August 2020, as evidenced by increases in the clustering coefficient and decreases in the shortest path length over time. In fact, the cohesion achieved by the SALURBAL network is among the highest documented in the literature. For example, Long et al.^17^ used social network analysis to characterize a complex translational cancer research network including collaborations between academic researchers and medical practitioners in Australia. This network reported clustering coefficients of 0.492 and 0.106 for past and current collaborations between network members, respectively, compared to the SALURBAL network, which was characterized by much higher clustering coefficients, ranging from 0.83 to 0.94. Another study described the evolution of the collaborative projects launched during the four years of implementation of the European Framework Programmes for research and innovation^18^. The shortest path length of these projects, range between 2.79 and 2.81, substantially longer than what we documented for the SALURBAL network (1.58–1.81). This difference could be an artifact of the SALURBAL network’s overall size, which is substantially smaller than the European network. Practically speaking, this could signal greater efficiency as the average member of the SALURBAL network can reach any other member in the network via just two, instead of three, other people. This high degree of connectivity means that there are multiple paths between network members, enabling effective and efficient diffusion of information and knowledge through the network. Cohesive collaboration networks have been found to foster the exchange of ideas, perspectives, and approaches to problem-solving which can facilitate generalized consensus among participants and innovative solutions^9,19^.

Diversity has been shown to improve productivity, spur innovation, enhance robustness, produce collective knowledge, and sustain further diversity^14^. Our analyses suggest that the SALURBAL network is highly diverse, and that this diversity extends within and across project activities, and to the whole project. Moreover, we observed diversity across attributes, including gender, career stage, discipline, sector, research topic, and geographic context (country and city). In other studies, this balance in the diversity of different attributes and across activities has been shown to reduce inefficiency and a level of complexity that is unmanageable and destructive to the project^14^. The diversity observed within and between project activities evolved purposefully and through governance and organizational structures designed to support it. Over the course of the project and since its inception, different activities were planned to maximize different forms of diversity while others were designed to cater to specific types of group members (such as junior researchers). For example, the GMB workshops were designed to generate intersectoral collaboration, by engaging with policymakers and other non-academic stakeholders in the research process in a way that influences the direction of the project. SALURBAL has partnerships with the InterAmerican Development Bank, the World Resources Institute, and the Pan American Health Organization, among others. The project’s success has been highly dependent on researchers networking with government officials to gain access to necessary data and data-gathering opportunities. The academic training workshops were planned to support diversity across levels of career stage in order to promote capacity building and as a training activity^7^. On the other hand, papers and proposals emerged more organically within the network, as lead authors select their co-authors with both autonomy and guidance from senior authors. This structure of SALURBAL activities added new diverse collaborations to the existing diverse collaborations that were maintained across activities, ultimately leading to the growth of the network.

The geographic diversity represented in the SALURBAL network is particularly important and novel. While several public health research networks in Latin America have been established in the last decade, they have included researchers from relatively few countries, tasked with investigating very specific topics^20^. For example, the GUIA (Guide for Useful Interventions for Physical Activity in Brazil and Latin America) project promotes physical activity with the participation of researchers from Brazil, the USA, and Colombia^21^, and the Collaborative Actions for Risk Factor Prevention and Effective Management of Noncommunicable Diseases (CARMEN), an initiative of the Pan American Health Organization, was established to reduce noncommunicable disease (NCD) risk factors through the participation of 14 Latin American countries, 7 Caribbean countries and Canada^22^. In contrast, the SALURBAL Project has among the most geographically diverse urban health collaboration networks, with participants from 26 countries and spanning some 24 disciplines. By bringing together stakeholders from across Latin America, the project and its many outputs contribute perspectives and knowledge from low- and middle-income countries which remain underrepresented within urban health^23^.

The project fostered interdisciplinary and intersectoral collaborations by encouraging the participation of diverse stakeholders and brokered regular in-person and remote interactions centered around numerous activities including, forums, symposia, and GMB workshops. This approach created opportunities for diverse stakeholders to meet and a platform for the development of sustained partnerships. Previous analysis of multisectoral and intersectoral initiatives in 36 Member States of the WHO European Region showed that intersectoral approaches form a critical component in the production of coherent and sustainable policies^24^.

The SALURBAL Project’s openness to engagement supported the inclusion of researchers across levels of career stage and gender. This diversity was observed both within and across the project’s activities and increased over time. The gender diversity achieved within SALURBAL represents an important achievement as international research collaborations tend to be gendered, with lower prevalence of international collaboration reported among women in academia compared to men^25^. According to a recent review, equitable gender representation in teams improves collaborations and is associated with increased collective intelligence (collective intelligence describes a team’s capacity to dynamically shift and respond to each other in light of different situations and pressures to maintain consistent outcome quality)^26^. Furthermore, gender-balanced collaborations foster more assertive science and innovation^27^. Diversity across levels of career stage within and across project activities provides unique opportunities to build research capacity^7^. It has been shown that mentoring processes and team-level support improve individual performance and satisfaction^28,29^.

There were some limitations in these analyses. First, participant attributes were not all self-reported. While some self-reported data was available from SALURBAL surveys and the project directory, other data needed to be sourced using online sources. For example, gender was not self-reported by the network participants, thus limiting the description to a binary classification based on the names of those who participated in each activity, a major limitation. Furthermore, we had to assign participants working across multiple disciplines, research topics and/ or sectors to just one category. We verified these cases with members of the SALURBAL executive committee who knew participants personally and were able to assist in the classification process. Another major limitation is that we were unable to assess diversity across race and ethnic identification and socioeconomic background. Future work needs to investigate and enhance diversity across these domains as they are critical to the goals of an inclusive science, to addressing past harms and exclusions and to the generation of valid and impactful science^30^. Second, in each institution there are other interactions, with actors and institutions, that were established through the participation in SALUBAL but that were not reported or made visible in this study. However, these “invisible” interactions enrich and strengthen the network over time. Third, while we discuss the potential implications of the governance and organizational structures of SALURBAL and their likely impact on the cohesion of the network and the diversity observed within and between activities, it is unclear to what extent these design features impacted the characteristics of the network and its changes over time. The strengths of this study include the use of a temporal network analysis framework and novel measures of cohesion and diversity to characterize the evolution of a complex collaboration framework and its activities.

Our results highlight the feasibility of creating research networks that are cohesive and diverse across multiple countries and institutions. Although the determinants of SALURBAL’s success in terms of cohesion and diversity (at least along the dimensions assessed) cannot be inferred from these results alone, we propose that several features of the SALURBAL organizational structure were likely important. These likely include: (1) the project was deliberately designed to encompass a diverse geographically and interdisciplinary network to capture the complexity of urban health in Latin America, (2) governance and administration structures supported engagement and participation across various dimensions and specifically implemented structures and activities designed with the explicit goals of capacity building (i.e., academic training workshops), team-building (i.e., person and virtual meetings) and engagement with non-academic partners (i.e., group model building, forums and symposia), disseminate research (i.e., papers, forums and symposia), (3) regular project activities were carried out virtually, making intensive use of online platforms, combined with biannual in-person team meetings, with all activities including senior investigators, junior investigators, and staff members increasing the cohesion, (4) the affiliate institutions were leaders in the region and had autonomy to put together their local teams, (5) The SALURBAL diversity is an ongoing characteristic that started from the LAC-Urban Health network, and (6) SALURBAL has made a special effort to be open to the incorporation of new participants, topics, methods and products. Given the scientific challenges facing our world today the creation and sustainability of diverse and cohesive research networks is fundamental to both generate and use scientific knowledge to protect population health and promote equity and environmental sustainability.

## Methods

### The SALURBAL project

The Salud Urbana en América Latina (SALURBAL) project is a novel international partnership for actionable evidence on urban health in Latin America^7^. While formally funded in 2017, this partnership has been evolving since 2015 as a network of researchers and policymakers across different countries who created the Urban Health Network for Latin America and the Caribbean (LAC-Urban Health)^7^. SALURBAL has a governance and operating structure that facilitates this collaboration, described in more detail elsewhere^7^ (see supplementary information, Figs. S1 and S2). The project team was initially convened by a small but geographically diverse group of researchers who sought to create a diverse and interdisciplinary team^31^. The project has contributed a range of outputs including academic and policymaker workshops, forums, academic papers, and policy briefs directly relevant to the research and policy landscape in Latin America (Supplementary information, Table S1). SALURBAL has analyzed and harmonized data relevant to urban health across a range of sources for all cities of 100,000 residents or more across 11 countries (n = 371) and, as of the beginning of the project until the time of this study (May 2017 to August 2020) 49 papers had been published.

### Data collection and study variables

Data collection for this study included two phases. First, we harmonized multiple sources of data from records administered by the SALURBAL Project coordinator regarding the project’s collaboration activities from May 2017 to August 2020. The data spanned multiple SALURBAL activities including 126 research proposals, three academic training workshops, monthly meetings of three Project Cores (the Data and Methods Core, the Built and Physical Environment Core, and the Social Environment Core) and 12 working groups, seven biannual project meetings, three GMB workshops, 49 papers approved or published, two policy symposia, and one Knowledge-to-Policy Forum. Second, we obtained individual-level information across seven attributes (country, city, discipline, research topic, sector, career stage, gender) from project registration forms and the SALURBAL directory. When the relevant information was not reported, or the information provided did not fit our classification of attributes (see Table S2 in the Supplement), we searched publicly available online records, including professional websites with biographical descriptions, published documents and workplace-specific websites. In cases where a participant worked across multiple sectors or disciplines, we assigned them to the sector or discipline in which they worked most of the time. We verified all information collected about participants with a member of the SALURBAL Project executive committee and a key team member who were familiar with participants. Country and city were defined according to the participants main work location (Supplementary information, Table S2). Discipline was defined according to the characteristics outlined by Krishnan^32^ (Supplementary information, Table S2). Research topic was defined as a subcategory of discipline, with categories outlined in Table S2 in the supplementary information document^33^. The sector was defined as public and government, academia, civic society, private sector, or intersectoral according to the sector of their primary place of employment. Career stage was defined according to the participants’ position title at their primary place of employment and their academic qualification (i.e., junior or senior, where junior was defined as student or individual with less than five years of experience). Gender was inferred based on available biographical information (women and men).

### Data analysis

We characterized the SALURBAL collaboration network using a temporal multilayer network approach. In this network, each layer represented a project activity type while the temporal window was defined using a Time Windows in Networks algorithm^34^. Once defined, the same temporal window was used for all measures. Then, we assessed the SALURBAL network cohesion over time using four structural property measures of the network, including density, average clustering coefficient, and average shortest path. To characterize diversity, we first created a measure of diversity *within* layers. Then, we validated the diversity scores using a configuration model to ensure that the scores were significantly higher than would otherwise be expected by chance. Second, we measured the diversity *between* layers using an adapted measure of diversity in multiplex networks by Carpi et al^35^. We also assessed the contribution of different SALURBAL activities to network diversity *between* activities using a layer-reduction method. Third, we assessed network diversity *within* and *between* the SALURBAL activities over time. We conducted multiple linear regression to determine what project activities were associated with more diversity. Lastly, we used a Louvain community detection algorithm to identify collaboration communities within each temporal window. 

### Characterization of the SALURBAL collaboration network

We characterized the evolving network of the SALURBAL Project participants and their collaborations from May 2017 to August 2020, using a temporal multilayer network approach. We define participants as individuals engaged in at least one SALURBAL Project activity during the studied time period. The project activities are grouped using a multiplex network structure divided into six undirected layers. Each layer comprised all the SALURBAL Project participants (nodes) engaged in the corresponding activity. Each node was characterized by seven attributes (country, city, discipline, research topic, sector, career stage, gender). The edges (or connections) between nodes represent collaborations between participants. Each edge had a weight that represents the number of collaborations between a pair of connected nodes in that particular layer.

We used a temporal network approach to evaluate the project’s diversity and cohesion over time. To define the appropriate temporal window size $w$, we aggregated our multiplex network into one layer. Then, we implemented a Time Windows in Networks (TWIN) algorithm that optimizes the trade-off between the noise and information contained in the data^34^. We divided the timespan of the project i.e., 40 months (May 2017 to August 2020), into different temporal windows $w$. For each temporal windows $w$, we calculated a range of network measures to analyze the network’s structural properties and the effect of the window size. These measures included: the number of connected components, the diameter which is the longest of all the shortest distance between all the possible nodes pairs, the average shortest path which is the average number of steps along the shortest path for all possible collaborations between participants, the radius which is the minimum of all the shortest distance between all the possible nodes pairs, the size of the largest clique which is defined as the maximum number of participants in a group when each member is connected to each of the others and the giant connected component which is the size of the largest connected component^15^. We also calculated the diversity *within* layers described above for each attribute to evaluate the diversity of SALURBAL participants’ collaborations with respect to their attributes. Using these measures, we constructed the statistical time series ${F}_{w}$ for each metric and window size. Then, we measured the noise by calculating the variance ${V(F}_{w}),$ and estimated the loss of information by calculating the compression ratio $R\left({F}_{w}\right)$. We defined the optimal temporal window $w$ that minimizes the absolute difference between ${V(F}_{w})$ and ${R(F}_{w})$ for each network measure. Finally, we averaged the optimal window of all the measures in order to choose the window size with the largest changes in the diversity and network structure^34^.

### Measures

#### Diversity *within* layers (project activities)

Let ${DW}_{l}^{o}$ be the diversity of each activity $l$ of the project, for each attribute $o\in O:\{discipline,domain,sector,gender,seniority,country,city\}$ of SALURBAL participant $i$. First, we evaluated the diversity of each node individually. This was done using a two-part process: (1) The intra-nodal process, which examines the dyads (direct connections) of the node $i$, and (2) The inter-nodal process, in which we defined subgraphs representing the working groups in which node $i$ participates, and we examined all possible connections of the nodes belonging to each subgraph. These two measures were multiplied by $\beta$ and $\left(1-\beta \right)$, respectively. As seen below:

1 $${DW}_{i}^{o,l}=\beta *\left( \frac{1}{{|v}_{i}^{l}|} \sum_{j\in {v}_{i}^{l}}\left(1- {\delta \left({x}_{i,}{x}_{j}\right)}^{o,l}\right) \right)+(1-\beta )\boldsymbol{*}\left( \frac{1}{{|v}_{i}^{l}|\left(\left|{v}_{i}^{l}\right|-1\right)}\sum_{j,k \in {v}_{i}^{l}}\left(1- {\delta \left({x}_{j,}{x}_{k}\right)}^{o,l}\right) \right)$$

where, ${v}_{i}$ is the neighborhood of node $i,{v}_{i}=\{i,j,\dots ,n\}$, ${x}_{i}$, ${x}_{j}$ and ${x}_{k}$ are the attribute values according to the outcome $o$ of the node $i,j,$ and $k,$ respectively. ${\delta \left({x}_{i,}{x}_{j}\right)}^{o,l}$ is a Kronecker delta that is equal to one if ${x}_{i}={x}_{j}$ or zero, otherwise.

Second, we calculated the diversity ${DW}_{l}^{o}$ of each layer $l$, as the average of ${DW}_{i}^{o,l}$ over all the nodes. The result ranges from 0 to 1; the closer the diversity score is to 1, the higher the diversity of the node $i$ or the layer $l$ of the SALURBAL network.

Lastly, we constructed a configuration model which is a random graph that preserves the degree sequence of participants' attributes *within* the SALURBAL network^16^. The nodes are randomly connected while ensuring that every attribute category in the network maintains the degree observed in the SALURBAL network, with a tolerance of 10%. We simulated a total of 1000 random graphs and calculated the diversity *within* layers for the successful simulations. Next, we calculated the ratio of the SALURBAL diversity to the simulated diversity (i.e., diversity observed in the SALURBAL network divided by the diversity observed across the simulated random graphs) and performed a Fischer p-value test for non-symmetrical distributions. A p-value lower than 0.1 was interpreted as greater diversity than would be expected by chance.

#### Diversity *between* layers (project activities)

While the diversity within layers allows us to quantify the diverse collaboration among the project participants in each project’s activity, it does not provide information on whether the project structure (i.e. project activities) is creating new diverse collaborations. We assessed the extent to which the diversity in collaborations differs *between* SALURBAL activities as characterized within the multiplex network. Each layer (i.e., project activity) is compared to the others, to assess the differences between the connectivity path (i.e., the differences in how a participant collaborates in one activity with some participants and in another activity with different participants than the first ones) in a way that allows us to evaluate the contribution of each activity in creating new and diverse collaborations. First, we calculated for each node $i$ the Node Distance Distribution ${N}_{i}^{\overline{p}}$ that specifies in a probabilistic manner the shortest path distance between node $i$ and all other nodes connecting to it in the same layer $\overline{p}$. The Transition Matrix ${T}_{i}^{\overline{p}}$ is the probability that node $i$ in layer $\overline{p}$ is reached in one step by a random walker^35^. Second, using these distributions, we calculated the node difference, that quantifies the differences of the connectivity paths of node $i$ in layers $\overline{p}$ and $\overline{q}$^35^, with the following equation:

2 $${D}_{i}\left(\overline{p},\overline{q}\right)=min\frac{\sqrt{J\left({N}_{i}^{\overline{p}},{N}_{i}^{\overline{q}}\right)}+\sqrt{J\left({T}_{i}^{\overline{p}},{T}_{i}^{\overline{q}}\right)}}{2\sqrt{log\left(2\right)}}$$

where, $J$ is the Jensen-Shannon (JS) divergence that measures the distance between two probability distributions. Third, we calculated the difference between layers as the average of ${D}_{i}\left(\overline{p},\overline{q}\right)$ over all the nodes. With this definition,${D}_{i}\left(\overline{p},\overline{q}\right)=0$ indicates that layers $p$ and $q$ are identical, while ${D}_{i}\left(\overline{p},\overline{q}\right)=1$ indicates that one of the layers is fully connected, while the other is totally disconnected^35^.

Finally, we defined global diversity recursively as $U\left(S\right)=\underset{{\overline{s}}_{i}\epsilon S}{max}\{U\left(S{s}_{i}\right)+D\left(\overline{{s}_{i}},S/\overline{{s}_{i}}\right)\}$ for all $S\in \widetilde{S}$ with $\left|S\right|\ge 2$ where $\left|S\right|$ represents the cardinality of the set of layers^35^. This layer reduction method is realized through dynamic programming, which can be explained in two steps: first, we selected the smallest $LD\left(\overline{p},\overline{q}\right)$ value and add it to the global diversity $U\left(S\right)$. Second, we removed the LD combinations which contribute least to system diversity $U\left(S\right)$.

To calculate the diversity of the participant’s attributes, we modified the adjacency matrix of the network ${A\left(i,j\right)}^{o,\overline{p}}=1-{\delta \left({x}_{i},{x}_{j}\right)}^{o,\overline{p}}$, only considering the diverse collaborations for the attribute $o$ in the layer $l$.

3 $$\mathrm{DB}=\frac{2*U\left(S\right)}{\left|S\right|-1}$$

To compare the diversity *within* layers and *between* layers over time, we standardized the results of the diversity *between* layers. First, we standardized the results by dividing by the number of layers because some activities were not performed in all the time windows. Second, to ensure the two measurements have the same objective maximization, we standardized the diversity *between* layers again, since a balance between new and maintained collaborations is desired to ensure long-term sustainability and growth of the SALURBAL network (Eq. 3). With this standardization, the diversity *between* layers ranges from zero to two. A value close to one for a given attribute indicates that around half the diverse collaborations are new and unique, while the other half are the same across layers (i.e., the same people collaborate in two or more project activities).

#### Assessing the diversity of SALURBAL network over time

With the optimal temporal window defined, we calculated the diversity *within* and *between* layers for each temporal window. Then, we analyzed the results of the diversity *between* layers and *within* layers by conducting a multiple linear regression to determine what project activities are associated with greater network diversity. The dependent variable is the average of the diversity *within* and *between* layers, and the independent variables are binary variables for each project activity, that is equal to one if the activity was realized in that time window or zero, otherwise. Lastly, we identified the communities (group of individuals that are strongly connected) for each temporal window using the Louvain community detection algorithm^16^. This algorithm detects the existence of clusters by optimizing the modularity for each community. The modularity quantifies the strength of a community by comparing the actual density of edges in a subgraph to the density one would expect to have in the subgraph if the vertices of the graph were attached regardless of community structure^16^. For each community, we formed a subgraph of the aggregated network of SALURBAL collaborations. Then, calculated the diversity *within* layers in each community (subgraph). The aim is to understand whether communities are constituted by diverse participants or whether the greatest connectivity exists among participants with the same attributes.

### Supplementary Information

Supplementary Information.

## Data Availability

The datasets generated during and/or analysed during the current study are available from the corresponding author on reasonable request.
