# Author Correction: Assessing cohesion and diversity in the collaboration network of the SALURBAL project

**DOI:** 10.1038/s41598-023-40860-9

**Published:** 2023-08-22

**Authors:** Sofía Baquero, Felipe Montes, Ivana Stankov, Olga L. Sarmiento, Pablo Medina, S. Claire Slesinski, Francisco Diez-Canseco, Maria F. Kroker-Lobos, Waleska Teixeira Caiaffa, Alejandra Vives, Marcio Alazraqui, Tonatiuh Barrientos-Gutiérrez, Ana V. Diez Roux

**Affiliations:** 1 Department of Industrial Engineering, Social and Health Complexity Center, Universidad de los Andes, Crr 1 Este No.19ª-40 Piso 8, 111711 Bogotá, Colombia; 2 Urban Health Collaborative, Dornsife School of Public Health, Drexel University, Philadelphia, PA 19104 USA; 3 UniSA Allied Health and Human Performance, University of South Australia, Adelaide, 5000 Australia; 4 School of Medicine, Universidad de los Andes, 111711 Bogotá, Colombia; 5 Pettenkofer School of Public Health, Ludwig-Maximilians-Universität München, 81377 Munich, Germany; 6 CRONICAS Centre of Excellence in Chronic Diseases, Universidad Peruana Cayetano Heredia, Lima, 15074 Peru; 7 INCAP Research Center for the Prevention of Chronic Diseases (CIIPEC), Institute of Nutrition of Central America and Panama (INCAP), Guatemala City, 01011 Guatemala; 8 Observatory for Urban Health in Belo Horizonte (OSUBH), Universidade Federal de Minas Gerais, Brazil, Belo Horizonte, MG 30130-100 Brazil; 9 Department of Public Health, CEDEUS, Universidad Católica de Chile, 8330077 Santiago, Chile; 10 Institute of Collective Health, National University of Lanús, Buenos Aires, Argentina; 11 Center for Population Health Research, National Institute of Public Health, 62100 Cuernavaca, Mexico

Correction to: *Scientific Reports* 10.1038/s41598-023-33641-x, published online 10 May 2023

The original version of this Article contained an error in the name of author Waleska Teixeira Caiaffa which was incorrectly given as Waleska Teixeira.

The original Article has been corrected.

