# Supplementary material for: Assessing cohesion and diversity in the collaboration network of the SALURBAL project
Source: Sci Rep. 2023 May 10;13:7590. doi: 10.1038/s41598-023-33641-x (PMC10172186; doi:10.1038/s41598-023-33641-x)
Supplement: Supplementary file 1 — Supplementary Information. [file 41598_2023_33641_MOESM1_ESM.docx]

**Supplementary Information for Assessing cohesion and diversity in the collaboration network of the SALURBAL project**

Sofía Baquero^a*^, Felipe Montes^a^, Ivana Stankov^b,c^, Olga L. Sarmiento^d^, Pablo Medina^a^, S. Claire Slesinski^e^, Francisco Diez-Canseco^f^, Maria F. Kroker-Lobos^g^, Waleska Teixeira^h^, Alejandra Vives^i^, Marcio Alazraqui^j^, Tonatiuh Barrientos-Gutiérrez^k^ and Ana V. Diez Roux^b^

*Sofía Baquero, Address: Crr 1 Este No.19A - 40 Piso 8, Bogotá, Colombia, Phone: +57 3167525836

Email: sdc.baquero10@uniandes.edu.co

Supplementary Information


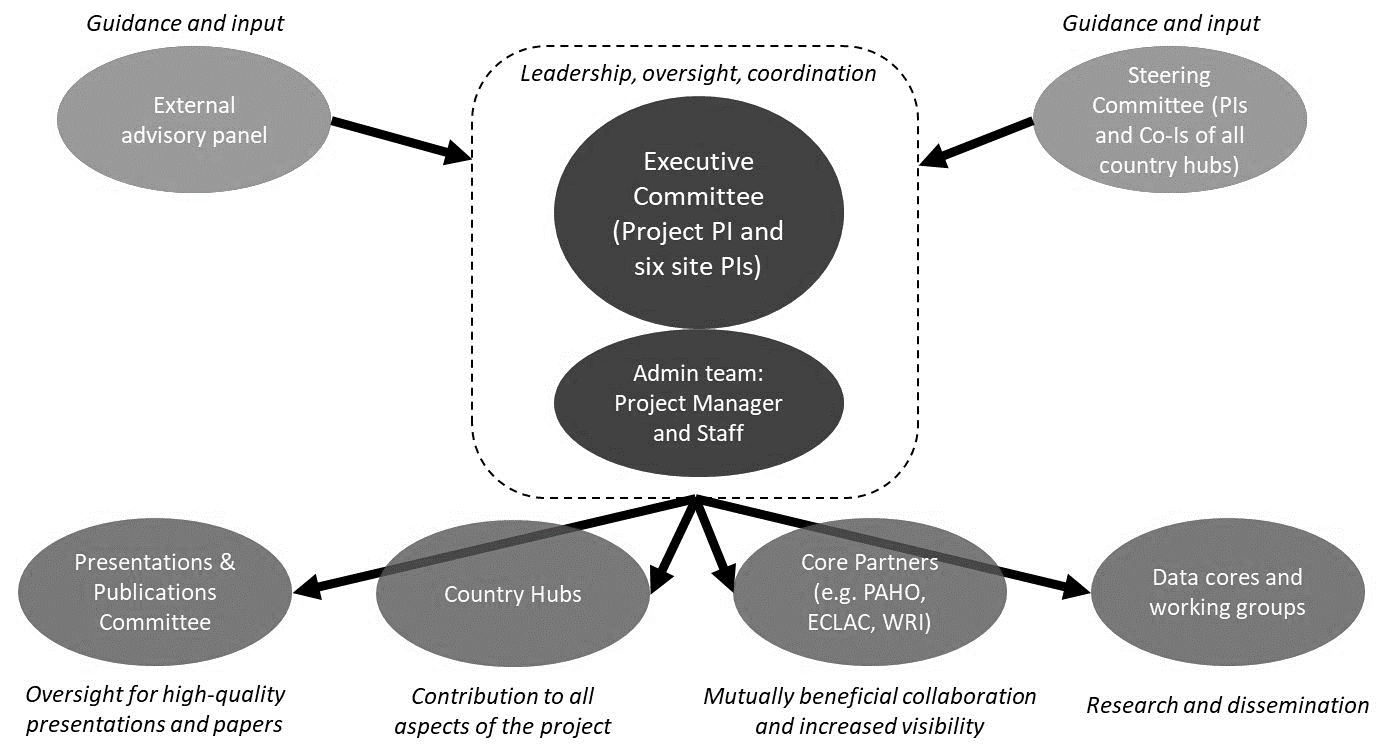


Fig. S1. SALURBAL’s Governance and Administrative Structure^1^.


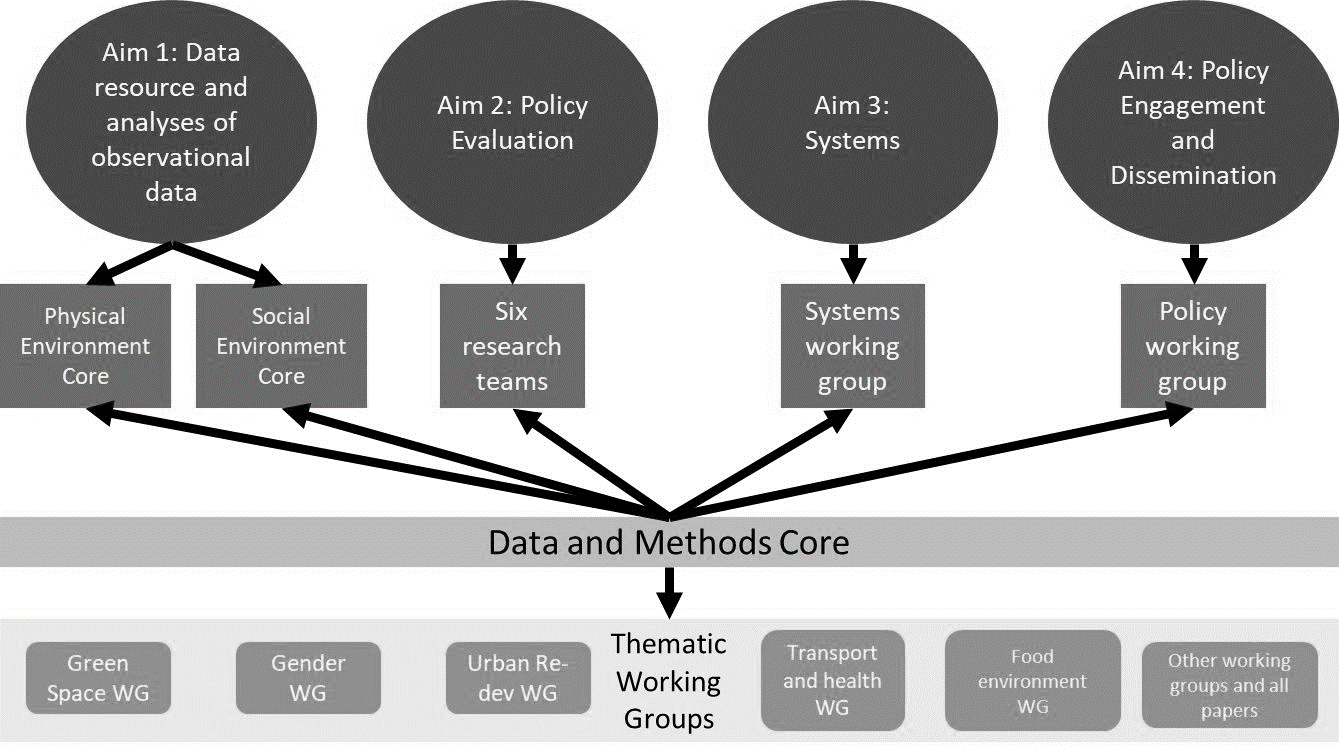


Fig. S2. SALURBAL’s Cores and Working Groups by Study Aims ^1^.

Table S1. SALURBAL project activities.

| Activity | Description | Goals | Frequency |
| --- | --- | --- | --- |
| Proposals | Manuscript proposals for peer review papers. The Publications and Presentations Committee (P&P) reviews proposals for publications and presentations. |  | Monthly |
| Academic training workshops | Multilevel analysis training workshops and Introduction to Directed Acyclic Graphs (DAGs) for Causal Inference workshop mainly for young investigators. | Capacity building for analyzing SALURBAL data. | Three: Belo Horizonte 2018, Lima 2019, and virtual 2020 |
| Meetings | Virtual full team meetings. Young and senior investigators are invited to present updates on the research. | Team meetings which to give presentations on their work at SALURBAL’s to receive input and support from the project’s senior research leadership and for the SALURBAL team to learn from each other. | Monthly |
|  | In-person team meetings in SALURBAL’s country hubs. During the two-day event. | These team meetings include updates on project policies and events but focus mostly on the presentation of “works-in-progress”, updates on and discussions of SALURBAL analyses and papers. SALURBAL team members facilitated a set of activities focused on dialogue and group reflection. | Biannual before COVID-19 pandemic |
|  | Virtual core and working group meetings (n:13). | Cores and working groups to move forward with tasks related to data compilation, analysis, creation of indicators, and interest groups. | Bimonthly to monthly |
| Group Model Building | Three in-person regional workshops (Lima, Guatemala y Brazil) with a diverse set of stakeholders (government, nonprofits, community members, international organizations, academics) that were very carefully designed to maximize diversity across geography, gender, discipline, | Workshop goals include: (1) to engage diverse stakeholders in the SALURBAL project and vision; (2) to provide stakeholders with experience in the application of systems approaches to urban health problems, and (3) to obtain stakeholder input that will help identify and prioritize research questions to be pursued by the SALURBAL Project using systems modeling in the future. | Three: Lima 2017, Sao Paulo 2018, Antigua 2018 |
| Papers | Approved papers by Publications and Presentations Committee (P&P) or peer review published papers. | To disseminate SALURBAL research. | 2019 (n=28) to July 2020 (n=20) |
| Forums and symposiums | Knowledge to Policy Forum. Forum participants represented an interdisciplinary and diverse group of organizations with an interest in urban health, equity, and sustainability. Attendees included representatives from municipal governments, health secretariats, non-governmental organizations, and community-based organizations. | First, introduced the project to key regional stakeholders, built connections with them, and increased the project’s overall visibility. Second, the project solicited and received feedback from these stakeholders on the project’s research questions, policy engagement activities, and dissemination products to inform SALURBAL’s ongoing activities. Finally, provided a space for these stakeholders to discuss regional challenges to the translation of knowledge to policy and developed a set of potential solutions and approaches for overcoming those challenges. | Mexico City 2018 |
|  | Local Policy Symposiums after biannual research team meetings held by countries hubs. Attendees included representatives from municipal governments, health secretariats, non-governmental organizations, and community-based organizations. | To discuss SALURBAL results in the context of local policies. | Bogotá 2017, Lima 2017, Antigua 2018 and Belo Horizonte 2019 |

Table S2. SALURBAL participants attributes.

| **Country** | **City** | **Discipline*** | **Research topic** | **Sector** | **Career stage** | **Gender** |
| --- | --- | --- | --- | --- | --- | --- |
| Argentina | Ames | Administration | Administrative Support | Academia | Senior | Women |
| Australia | Ann Arbor | Anthropology | Aging | Intersectoral | Junior | Men |
| Bolivia | Austin | Architecture | Air Pollution | Private Sector | |  |
| Brazil | Barcelona | Communications | Biostatistics | Public and Government | |  |
| Canada | Barranquilla | Complex Systems | Built Environment |  |  |  |
| Chile | Beijing | Computer Science | Data Analysis |  |  |  |
| China | Belo Horizonte | Data Management | Data Science |  |  |  |
| Colombia | Berkeley | Demography | Data Visualization |  |  |  |
| Costa Rica | Bethesda | Economics | Demographic Transition |  |  |  |
| Ecuador | Bogotá | Engineering | Dissemination |  |  |  |
| El Salvador | Boston | Environmental Health | Econometrics |  |  |  |
| France | Braga | Environmental Sciences / Environmental Studies | Employment |  |  |  |
| Guatemala | Brasilia | Epidemiology | Financial Management |  |  |  |
| Honduras | Buenos Aires | Geography | Gender Disparities |  |  |  |
| Indonesia | Cali | Geophysics | Gender Statistics |  |  |  |
| Mexico | Cambridge | Management | GIS |  |  |  |
| Panama | Caracas | Public Health | Health Behaviors |  |  |  |
| Peru | Centre County | Public Policy | Health Communication |  |  |  |
| Portugal | Chapel Hill | Physics | Health Disparities |  |  |  |
| Saudi Arabia | Chicago | Social System | Health Economics |  |  |  |
| Singapore | Ciudad de Guatemala | Sociology | Health Management |  |  |  |
| Spain | Ciudad de Mexico | Statistics | Language Interpretation |  |  |  |
| Switzerland | Ciudad de Panamá | Systems Thinking | Maternal & Child Health |  |  |  |
| United Kingdom | Cochabomba |  | Mobility |  |  |  |
| USA | Córdoba |  | Mortality |  |  |  |
| Venezuela | Cuernavaca |  | Network Analysis |  |  |  |
|  | Cuiabá |  | Non-Communicable Diseases | |  |  |
|  | Diamantina |  | Nutrition |  |  |  |
|  | Durham |  | Obesity |  |  |  |
|  | Feira de Santana |  | Operations Research |  |  |  |
|  | Gainesville |  | Peri-Urban Studies |  |  |  |
|  | Geneva |  | Physical Activity |  |  |  |
|  | Goiania |  | Policy evaluation |  |  |  |
|  | Governador Valadares | | Policymaker Engagement |  |  |  |
|  | Ibagué |  | Project Administration |  |  |  |
|  | Jeddah |  | Public Management |  |  |  |
|  | Lanús |  | Qualitative Research |  |  |  |
|  | Lima |  | Remote Sensing |  |  |  |
|  | London |  | Road safety |  |  |  |
|  | Los Angeles |  | Segregation |  |  |  |
|  | Madison |  | Simulation-based Methods | |  |  |
|  | Makassar |  | Social Determinants of Health | |  |  |
|  | Medellín |  | Social Dynamics |  |  |  |
|  | Melbourne |  | Social Environment |  |  |  |
|  | Mexico City |  | Social Epidemiology |  |  |  |
|  | Minneapolis |  | Social Work |  |  |  |
|  | Montpellier |  | Spatial Analysis |  |  |  |
|  | Natal |  | Statistical Analysis |  |  |  |
|  | New Haven |  | Transport |  |  |  |
|  | New York |  | Urban Health |  |  |  |
|  | Ottawa |  | Urban Planning |  |  |  |
|  | Ouro Preto |  | Violence |  |  |  |
|  | Philadelphia |  |  |  |  |  |
|  | Porto |  |  |  |  |  |
|  | Quito |  |  |  |  |  |
|  | Raleigh |  |  |  |  |  |
|  | Rio de Janeiro |  |  |  |  |  |
|  | Salvador da Bahia |  |  |  |  |  |
|  | San José |  |  |  |  |  |
|  | San Salvador |  |  |  |  |  |
|  | Santiago de Chile |  |  |  |  |  |
|  | Santo Andre |  |  |  |  |  |
|  | São Paulo |  |  |  |  |  |
|  | Seattle |  |  |  |  |  |
|  | Singapore |  |  |  |  |  |
|  | St. Louis |  |  |  |  |  |
|  | Stanford |  |  |  |  |  |
|  | Tandil |  |  |  |  |  |
|  | Tegucigalpa |  |  |  |  |  |
|  | Valencia |  |  |  |  |  |
|  | Vancouver |  |  |  |  |  |
|  | Villa Nueva |  |  |  |  |  |
|  | Washington D.C. |  |  |  |  |  |

* To be considered a discipline, it must fulfill the following characteristics: have a particular object of research, have a body of accumulated specialist knowledge, have theories and concepts; use specific terminologies; have developed specific research methods; must have some institutional manifestation in the form of subjects taught and professional associations connected to it ^2^.

**SI References**

1. Slesinski, C., Roux, A. D. & SALURBAL team. *SALURBAL Mid-Project report 2019*. (2019).

2. Krishnan, A. What are academic disciplines? *Natl. Cent. Res. Methods* 57 (2009).
